# Depression in left-behind adolescents from single-parent families: a nomogram based on multidimensional risk factors

**DOI:** 10.1186/s13034-025-00894-5

**Published:** 2025-04-02

**Authors:** Wang-Cheng Cen, Cheng-Han Li, Wen-Jing Yan, Yu-Qi Sun

**Affiliations:** 1 School of Mental Health, Wenzhou Medical University, Wenzhou, 325000 China; 2 Third Affiliated Hospital of Wenzhou Medical University, Wenzhou, China

**Keywords:** Single-parent left-behind adolescents, Depression, Nomogram, Lasso regression, Mental health screening

## Abstract

**Background:**

Depression is a significant issue affecting adolescents’ mental health. While depression research is relatively extensive, studies focusing on left-behind adolescents from single-parent families remain limited. Due to their unique family structure, this group is more susceptible to multiple stressors, increasing their risk of depression.

**Objective:**

This study aims to construct a predictive model based on a nomogram to identify the multidimensional characteristics of depression risk among left-behind adolescents from single-parent families, providing theoretical and practical evidence for early screening and targeted mental health interventions.

**Methods:**

Cross-sectional data from the China Psychological Health Guardian Project (CPHG) were utilized to select samples of left-behind adolescents aged 12–20 years from single-parent families (*N* = 3731). Key variables were identified using Lasso regression, followed by the optimization of the model through binary logistic regression. A nomogram prediction model was then constructed based on significant variables.

**Results:**

The study identified gender, age, duration of parental separation, family satisfaction, parental education levels, substance dependence, weekday sleep duration, weekend mobile phone use duration, and chronic diseases as key predictors of depression risk. The nomogram model demonstrated good discriminatory and predictive accuracy, with AUC values of 0.771 and 0.759 in the training and validation sets, respectively.

**Conclusion:**

By integrating multidimensional variables, this study developed a predictive model for depression risk among left-behind adolescents from single-parent families, providing scientific evidence for the early identification and intervention of high-risk individuals.

## Introduction

Depression is a widespread and debilitating mental disorder characterized by persistent sadness, loss of interest or pleasure, sleep disturbances, fatigue, difficulty concentrating, and feelings of worthlessness [1]. As one of the most prevalent mental health issues among adolescents worldwide, its high incidence during this critical developmental stage profoundly impacts academic performance, interpersonal relationships, and overall quality of life [1, 2]. In recent years, the rising prevalence of depression among adolescents has become increasingly evident, particularly among those in special family environments, due to higher social pressures and different family structures [3]. Depression not only impairs the adolescents’ academic performance but also leads to social withdrawal and decreased life satisfaction, potentially hindering their future development [4].

Depression is not only a clinical disorder but also a culturally shaped phenomenon, with its prevalence, symptom expression, and coping mechanisms varying across different sociocultural contexts [5]. In Western countries, depression is often associated with internalizing symptoms such as sadness and low self-esteem, whereas in East Asian cultures, individuals with depression may exhibit more somatic symptoms such as fatigue and headaches due to cultural stigma surrounding mental illness [5]. Family structure and societal expectations also play a crucial role in shaping adolescent mental health. In China, strong familial bonds and collectivist values emphasize family harmony, meaning that single-parent and left-behind adolescents may experience greater psychological stress due to deviations from traditional family structures [6]. Moreover, Chinese adolescents face intense academic pressures, which, coupled with limited parental support in single-parent households, may further elevate their risk of depression [7]. Given these cultural specificities, it is essential to consider how depression manifests in Chinese adolescents and whether existing risk models adequately capture these cultural influences.

Although adolescent depression has garnered significant research attention, existing studies primarily focus on general adolescent populations, with limited exploration of left-behind adolescents from single-parent families [8, 9]. These adolescents, due to the extended absence of one parent, often face a lack of emotional support, reduced stability in life, and increased stress [10, 11]. Literature indicates that these adolescents frequently experience parent-child relationship alienation, an increased economic burdens, and amplified uncertainty in their lives, all of which significantly elevate the risk of depression [12]. Therefore, identifying depression risk factors for left-behind adolescents from single-parent families and developing scientifically robust predictive models are of crucial clinical and social relevance for early intervention and mental health support.

In evaluating depression risk among left-behind adolescents from single-parent families, the integration of multidimensional variables provides a comprehensive understanding of their psychological challenges under compounded stress. The cumulative risk model emphasizes that individuals’ psychological vulnerability increases with the accumulation of adverse factors, especially in the absence of adequate support systems [13]. The concurrent challenges that left-behind adolescents from single-parent families often face include: lack of emotional support, increased economic burdens, and lifestyle restrictions [14]. The interaction and accumulation of these factors may intensify their depressive tendencies [14]. Integrating multidimensional variables not only captures the emotional risks faced by these adolescents in challenging life contexts but also lays a solid theoretical foundation for the development of accurate predictive models.

Under the framework of the cumulative risk model, demographic variables and family economic factors form the foundation of depression risk for left-behind adolescents from single-parent families [15]. Gender and age are significant influencing factors: studies have shown that female adolescents are more prone to depression during puberty, and older age often correlates with greater social pressures, exacerbating emotional challenges faced by left-behind adolescents [16]. Moreover, the extended absence of a parent leaves these adolescents with a lack of emotional support in parent-child relationships, compounded by the uncertainty of their living environment, placing them at a disadvantage in coping with stress [15]. Family economic instability not only increases life stress but also limits access to mental health support, creating a cumulative effect of multiple adverse factors [17]. These factors collectively amplify the psychological vulnerability of left-behind adolescents from single-parent families.

Health status and lifestyle factors also play critical roles in depression risk among left-behind adolescents [18, 19]. Chronic illnesses and substance dependence increase emotional instability, making adolescents more susceptible to negative emotions [19]. Additionally, sleep quality and smartphone usage habits significantly affect mental health [18, 20]: research indicates that sufficient sleep during weekdays and weekends promotes emotional recovery, whereas excessive smartphone use disrupts sleep, heightening emotional fluctuations and increasing depression risk [18]. Furthermore, engaging in regular physical activity has been shown to contribute to better emotional regulation among adolescents, providing both neurobiological and psychosocial benefits that support mental well-being and resilience against life stress [21]. The interplay of these health and lifestyle factors illustrates the cumulative stress effect ad described by the cumulative risk model, providing a profound perspective for fully understand the emotional risk characteristics of this group.

Despite existing studies on depression risk factors among adolescents, most focus on general populations, lacking in-depth analysis of the high-risk group of left-behind adolescents from single-parent families. Moreover, current depression risk models are often based on single or limited variables, failing to accurately reflect the emotional risks thet these adolescents face in contexts of compounded stress [22, 23].

Based on the above theoretical background, this study proposes the following hypothesis: among left-behind adolescents from single-parent families, multidimensional factors such as gender, age, family economic status, health status, and lifestyle significantly influence depression risk. In the absence of adequate support systems, these cumulative stressors will substantially amplify depressive tendencies. This study aims to integrate multidimensional variables to explore the major factors influencing depression risk and analyze the changes in risk trends when these factors accumulate, laying a solid theoretical foundation for developing accurate predictive models.

To achieve this goal, this study developed and applied a multidimensional nomogram-based predictive model, providing a scientific and intuitive tool for assessing depression risk. This method not only improves the model’s predictive accuracy but also proveides visual support for clinical and mental health screenings. It helps mental health professionals in the early identification of high-risk individuals and the formulation of more accurate intervention plans with significant clinical value and social relevance.

## Methods

### Study design and sample

This study used data from the China Psychological Health Guardian Project (CPHG) and employed a cross-sectional design to explore predictive factors of depression risk among single-parent adolescents aged 12–20 years. Data collection was conducted from October 2022 to May 2023, covering 569 collection sites in Nanchong, Sichuan Province, including schools, community care centers, and children’s hospitals. The project primarily focused on underrepresented child populations in social and psychological care systems, such as orphans, children without guardians, single-parent children, and left-behind children. The total dataset included 249,772 children.

For this study, a sample of single-parent adolescents was extracted from the CPHG database. The selection criteria included adolescents aged 12–20 years, exclusion of cases with missing values in any variable, and removal of samples with logically inconsistent questionnaire responses. Finally, 3731 eligible cases were included. Data collection was conducted through questionnaires, encompassing demographic variables such as gender, age, and family socioeconomic status, as well as variables like parental separation age, family satisfaction, and exercise frequency.

### Collection of data

#### Demographic variables

Demographic variables in this study included gender, age, and residential area. Gender was coded as a categorical variable (male = 1, female = 0). Age was recorded as a continuous variable, representing participants’ actual age. Residential area recorded the living environment, with urban areas coded as 1 and rural areas as 2.

#### Family and economic variables

*Parental separation experience*

The age at first parental separation was categorized into infancy (0–1.5 years), toddlerhood (1.5–3 years), preschool (3–6 years), school age (6–12 years), and adolescence (12–18 years). Duration of separation was categorized as “6 months to 1 year,” “1 to 2 years,” “2 to 4 years,” “5 to 10 years,” and “over 10 years.”

*Family satisfaction*

Family satisfaction measured participants’ overall satisfaction with family relationships using a five-point Likert scale ranging from “very satisfied” to “very dissatisfied.” This variable assessed the role of family relationships in predicting depression risk.

*Family socioeconomic status*

Subjective family economic status was rated on a four-point scale from “poor” to “wealthy.” Parental education levels were recorded as the highest level of education achieved, categorized into “vocational school or below,” “college,” “bachelor’s degree,” and “master’s degree or above.”

#### Health and lifestyle variables

Health and lifestyle variables included chronic illness, substance dependence, sleep duration, smartphone usage duration, and physical exercise. Chronic illness recorded whether participants had chronic diseases (no = 0, yes = 1). Substance dependence captured regular use of medications, including prescription drugs, over-the-counter medications, and supplements, coded as 0 (no) or 1 (yes), without implying addiction or misuse. Sleep duration was assessed separately for weekdays and weekends, categorized into “<5 h,” “5–6 h,” “7–8 h,” “9–10 h,” and “>10 h.” Smartphone usage during weekdays and weekends was recorded as average daily use, categorized into “never,” “<1 h,” “1–2 h,” “2–3 h,” “3–4 h,” and “>4 h.” Physical exercise frequency was categorized into “never,” “weekend-only,” “occasionally (1–2 times per week),” “frequently (3–4 times per week),” and “daily.”

#### Depression scale

The study employed the Center for Epidemiologic Studies Depression Scale (CES-D) to assess participants’ depressive symptoms. The scale consists of 20 items measuring the frequency of depressive symptoms in the past week. Each item is rated on a four-point Likert scale from “rarely” (0 points) to “almost always” (3 points). Total scores are calculated by summing all items, with scores above 16 indicating depressive symptoms (coded as 1) and scores ≤ 16 indicating no depressive symptoms (coded as 0). This classification standard has been widely applied [24]. The CES-D scale had a Cronbach’s alpha of 0.949 in this study, indicating excellent reliability and validity.

### Data analysis

Data analysis was conducted using RStudio 4.4.0. The dataset was randomly split into training (70%) and validation (30%) sets. Before conducting the analysis, a power analysis was performed using the pwr package in R. Based on an expected effect size (*Cohen’s f²* = 0.15), 15 predictors, a significance level of 0.05, and a desired power of 0.80. The calculated required sample size was 123. Both the training set (*N* = 2612) and the validation set (*N* = 1119) exceeded this requirement. The glmnet package was used for Lasso regression in the training set. Lasso regression, which incorporates an L1 penalty, effectively controls multicollinearity during variable selection and reduces model complexity to avoid overfitting. Compared to univariate logistic regression, Lasso regression enables simultaneous variable selection and shrinkage, resulting in a more robust model.

Significant variables identified through Lasso regression were further analyzed using multivariable binary logistic regression to optimize model interpretability and predictive accuracy. A nomogram model was developed based on the final selected variables. The model’s discrimination, calibration, and clinical utility were evaluated in both training and validation sets using ROC curves, calibration curves, and decision curve analysis (DCA).

## Results

### Baseline characteristics comparison between training and validation cohorts

As shown in Table 1, there were no statistically significant differences in baseline characteristics between the training and validation cohorts (*P* > 0.05).

**Table 1:** Comparison of variable characteristics between the training and validation datasets

Variables	Training dataset (*N* = 2612)	Validation dataset (*N* = 1119)	*P*
Age	14.05 ± 1.67	14.08 ± 1.69	0.636
Gender			
Female	1337 (51.2%)	555 (49.6%)	0.387
Male	1274 (48.8%)	564 (50.4%)	
Residence			
Urban	1024(51.8%)	407(49.3%)	0.258
Rural	952 (48.2%)	417 (50.7%)	
Depression			
Yes	1976 (75.7%)	824 (73.6%)	0.200
No	635 (24.3%)	295 (26.4%)	
Separation_age	3.18 ± 1.28	3.17 ± 1.3	0.823
Separation_duration	2.75 ± 1.4	2.75 ± 1.4	0.929
Family_satisfaction	3.59 ± 0.98	3.62 ± 1	0.321
Parental_education_level	1.19 ± 0.54	1.2 ± 0.53	0.660
Economic_status	1.93 ± 0.58	1.94 ± 0.58	0.587
Workingday_sleep	2.74 ± 0.74	2.74 ± 0.74	0.966
Weekend_sleep	3.37 ± 0.88	3.36 ± 0.89	0.761
Weekday_phone_time	1.98 ± 1.22	1.97 ± 1.24	0.671
Weekend_phone_time	3.91 ± 1.59	3.92 ± 1.59	0.887
Exercise_frequency	3.1 ± 1.11	3.05 ± 1.16	0.257
Drug_dependent			
Yes	2512 (96.2%)	1082 (96.7%)	0.529
No	99 (3.8%)	37 (3.3%)	
Chronic_disease			
Yes	2044 (78.3%)	875 (78.2%)	0.986
No	567 (21.7%)	244 (21.8%)	

### Variable selection using Lasso regression

**Fig. 1:**
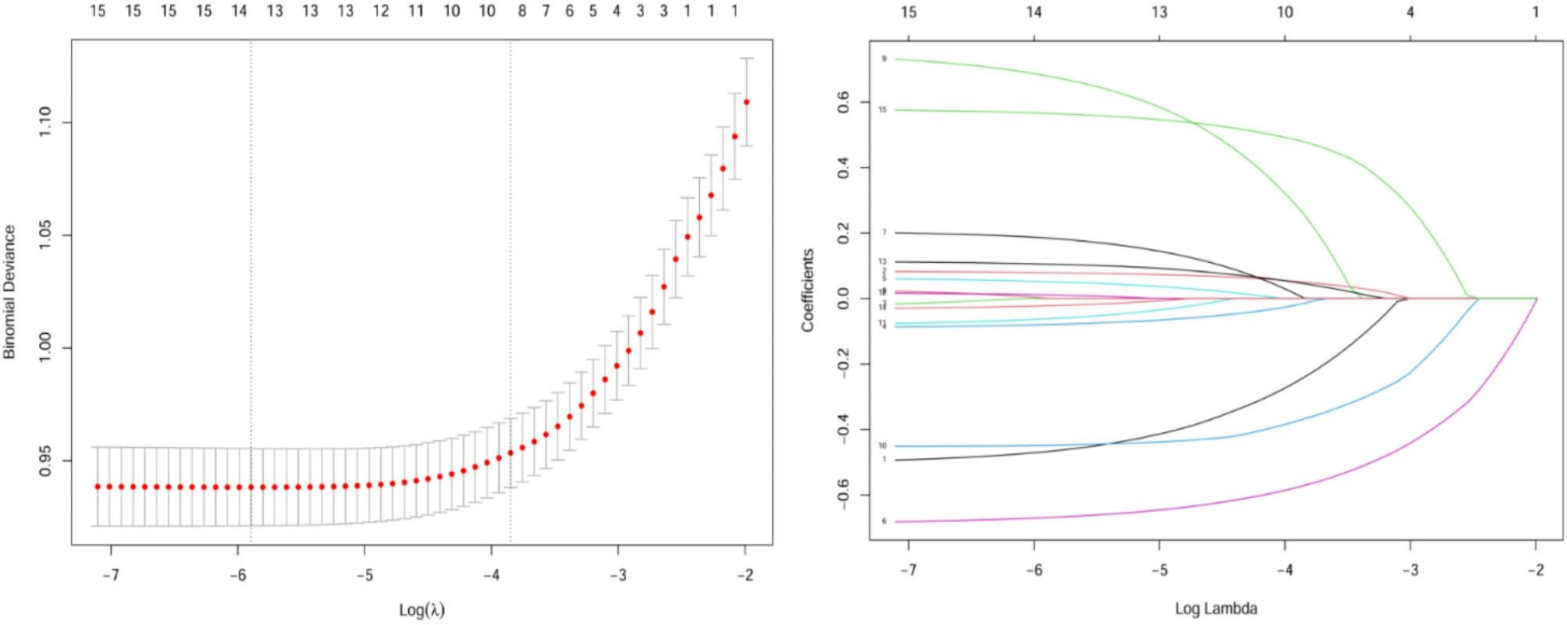
Selection of the optimal λ value for variable selection using Lasso regression (left) and coefficient shrinkage paths of predictors (right). The left panel displays the binomial deviance for different log(λ) values, where red dots represent the mean deviance, and the error bars indicate standard deviations. Two vertical dashed lines correspond to the λ values selected by the minimum mean squared error criterion (left line) and the one-standard-error rule (right line), respectively. The right panel illustrates the coefficient shrinkage paths of different predictors as log(λ) changes, with each line representing a variable. As λ increases, coefficients gradually shrink toward zero, and only significant predictors are retained at the optimal λ value

In the training cohort, depression status was used as the dependent variable, and all 15 candidate variables were included in the Lasso regression model for selection. Based on the optimal λ value (0.00331) from the plot, 14 variables with non-zero coefficients were identified, excluding the variable “age at parental separation” (Fig. 1). The selected 14 variables were subsequently used in the multivariable logistic regression analysis to further optimize the depression prediction model.

### Binary logistic regression analysis

Depression status (coded as no depression = 0, depression = 1) was set as the dependent variable, and the variables identified through Lasso regression were included as independent variables in the binary logistic regression analysis. Nine core variables were determined to be significantly associated with depression status, including gender, age, duration of separation, family satisfaction, parental education level, substance dependence, weekday sleep duration, weekend smartphone usage duration, and chronic illness (*P* < 0.05). Detailed results are provided in Table 2.

**Table 2:** Results of multivariate logistic regression

	OR	95% CI lower	95% CI upper	*P* value
Gender				
Female	Ref			
Male	0.604	0.492	0.742	< 0.001
Age	1.087	1.020	1.159	0.010
Residence				
Urban	Ref			
Rural	0.982	0.796	1.212	0.867
Separation_duration	1.087	1.012	1.167	0.022
Family_satisfaction	0.501	0.449	0.558	< 0.001
Parental_education_level	1.248	1.049	1.486	0.013
Economic_status	1.030	0.863	1.229	0.746
Drug_dependent				
Yes	Ref			
No	2.140	1.322	3.462	0.002
Workingday_sleep	0.638	0.542	0.750	< 0.001
Weekend_sleep	0.919	0.813	1.038	0.175
Weekday_phone_time	1.021	0.940	1.109	0.616
Weekend_phone_time	1.117	1.040	1.200	0.002
Exercise_frequency	0.967	0.883	1.060	0.480
Chronic_disease				
Yes	Ref			
No	1.797	1.431	2.256	< 0.001

### Construction of the nomogram prediction model

**Fig. 2:**
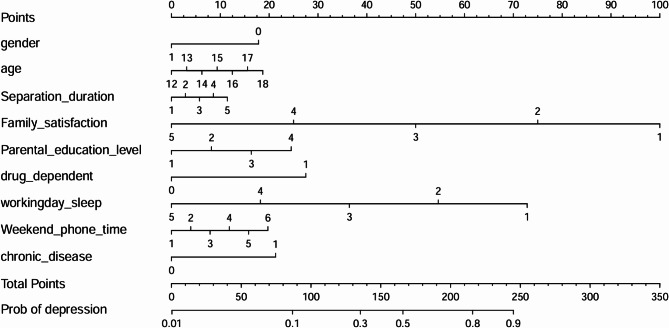
Nomogram depicting the predicted probability of depression risk based on key multidimensional factors, including demographic, family, health, and lifestyle variables

A nomogram prediction model for depression status was developed based on the key variables identified through Lasso regression and binary logistic regression analyses, including gender, age, duration of separation, family satisfaction, parental education level, substance dependence, weekday sleep duration, weekend smartphone usage duration, and chronic illness (Fig. 2).

The nomogram is used to predict the individual’s risk of depression. The process works as follows: First, the specific value of each variable for the subject is identified on its corresponding scale, and the value is projected upwards onto the “Points” axis to obtain the score for that variable. Next, the scores for all variables are summed to obtain the “Total Points.” Finally, the total score is located on the “Total Points” axis, and a vertical projection is made downwards to the “Prob of Depression” axis, which gives the predicted probability of depression for the individual.

### Validation of the nomogram model

**Fig. 3:**
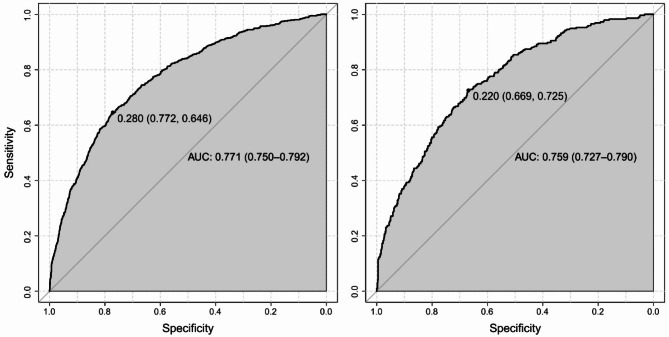
ROC curves for the training dataset (left) and validation dataset (right), illustrating the model’s discriminatory ability. The area under the curve (AUC) values are 0.771 (95% CI 0.750–0.792) for the training dataset and 0.759 (95% CI 0.727–0.790) for the validation dataset, indicating good predictive performance. The marked points on each curve represent the optimal threshold values, with corresponding sensitivity and specificity

**Fig. 4:**
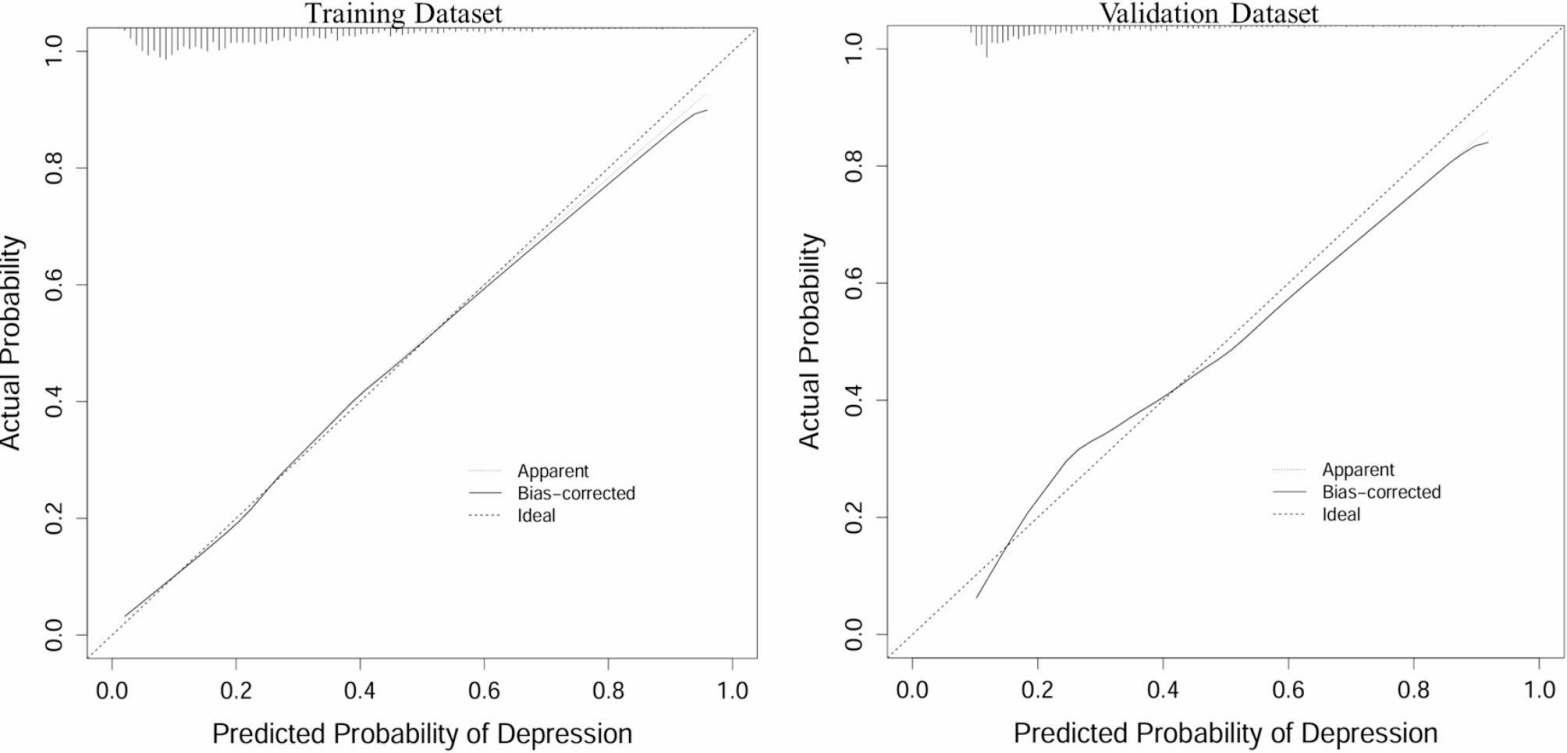
Calibration curves for the training dataset (left) and validation dataset (right), assessing the agreement between predicted and actual probabilities of depression. The “Ideal” line represents perfect calibration where predicted probabilities exactly match actual outcomes. The “Apparent” line shows the observed calibration, while the “Bias-corrected” line accounts for overfitting through internal validation. The close alignment of the “Bias-corrected” line with the “Ideal” line indicates that the model exhibits good calibration in both datasets

**Fig. 5:**
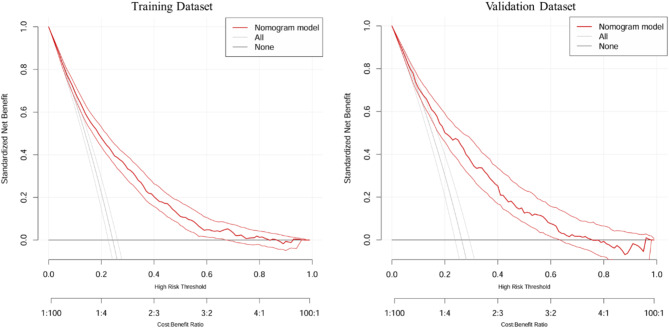
Decision curve analysis (DCA) for the training dataset (left) and validation dataset (right), demonstrating the net clinical benefit of the nomogram model across different risk thresholds. The “Nomogram model” curve represents the net benefit of using the proposed predictive model, while the “All” and “None” curves represent the strategies of treating all individuals as high risk or none as high risk, respectively. The higher net benefit of the “Nomogram model” across a wide range of risk thresholds suggests its clinical utility in identifying high-risk individuals more effectively than the alternative strategies

To comprehensively evaluate the predictive performance and stability of the nomogram model, three analyses were conducted: receiver operating characteristic (ROC) curve analysis to assess the model’s discriminatory ability (Fig. 3), calibration curve analysis to evaluate the agreement between predicted and observed outcomes (Fig. 4), and decision curve analysis (DCA) to assess the clinical utility of the model (Fig. 5).

In Fig. 3, the ROC curve shows the trade-off between sensitivity and specificity for the nomogram model. The areas under the curve (AUCs) were 0.771 (95% CI 0.750–0.792) in the training dataset and 0.759 (95% CI 0.727–0.790) in the validation dataset, indicating good discriminatory ability of the model. A higher AUC value reflects better capability of the model to distinguish between depressed and non-depressed individuals.

In Fig. 4, the calibration curves for the training and validation datasets align closely with the diagonal reference line, illustrating that the predicted probabilities of depression closely match the observed probabilities. This suggests the model has high predictive accuracy and minimal deviation.

In Fig. 5, the decision curve analysis (DCA) evaluates the net clinical benefit of the nomogram model at various risk thresholds. The model shows the highest net benefit compared to treating all or no individuals as depressed, particularly in the risk threshold range of 0.1–0.6. This indicates that the nomogram provides meaningful guidance for clinical decision-making by identifying individuals at high risk of depression, thereby enabling targeted interventions.

## Discussion

In this study, we systematically explored the predictive effects of several factors on depression risk among single-parent adolescents using cross-sectional data. The results identified gender, age, duration of separation, family satisfaction, parental education level, substance dependence, weekday sleep duration, weekend smartphone usage, and chronic illness as independent predictors of depression. Specifically, male gender, higher family satisfaction and parental education levels, and adequate weekday sleep duration were significantly associated with reduced depression risk. Otherwise, prolonged separation, substance dependence, extended weekend smartphone usage, and chronic illness were found to increase depression risk. These findings validate the cumulative risk model, highlighting the amplifying effect of multiple adverse factors on emotional vulnerability.

Firstly, family satisfaction was negatively associated with depression risk, consistent with previous studies [25, 26]. High family satisfaction typically reflects harmonious family relationships and emotional support, which enhance adolescents’ sense of belonging and help them cope with negative emotions [25]. In single-parent families, higher family satisfaction is particularly crucial as it partially compensates for the lack of dual-parent support, thereby reducing adolescents’ depression risk [26]. Secondly, extended parental separation significantly increased depression risk among single-parent adolescents, aligning with the core tenets of attachment theory, that emphasizes the importance of secure parent-child relationships in emotional regulation, and prolonged separation undermines such attachment, making it harder for adolescents to access emotional support in stressful situations [16, 27]. Research suggests that separation-induced disruption in parent-child relationships reduces opportunities for guidance and support, weakening emotional security and increasing depression risk [10, 16].

Substance dependence and chronic illness also played significant roles in depression risk among single-parent adolescents [19, 28–30]. According to stress coping theory, adolescents lacking effective coping strategies often resort to short-term emotion regulation behaviors, such as substance dependence, which can exacerbate emotional instability [29, 30]. Single-parent adolescents, deprived of coordinated parental support, are particularly vulnerable to emotional fluctuations caused by substance dependence. Chronic illness further compounds this vulnerability, imposing persistent physiological burdens and feelings of isolation [19]. For adolescents who rely on social support to alleviate emotional stress, chronic illness-induced social limitations and emotional isolation can intensify depressive tendencies. The interplay of these factors creates an emotional burden, undermining adolescents’ ability to cope with stress [28].

In addition, lifestyle factors, including weekday sleep duration and weekend smartphone usage, significantly influenced depression risk [18, 31]. Adequate weekday sleep promotes emotional recovery and psychological resilience, enabling adolescents to better regulate emotions under stress [18]. Conversely, excessive smartphone use during weekends may disrupt sleep, reduce the quality of social interactions, and exacerbate emotional issues [31]. In single-parent families, adolescents may rely more heavily on smartphones for social fulfillment, but such usage patterns could lead to sleep deprivation, diminishing their capacity to manage life stress. These findings underscore the importance of healthy lifestyle habits in emotional regulation; appropriate sleep and smartphone use enhance adolescents’ psychological resilience and stress management.

Parental education level was also found to have a protective effect against depression, likely due to the resources and coping strategies provided by families with higher education levels. Parents with higher education levels often possess greater mental health awareness and can promptly identify and address their children’s emotional issues, providing rational support [17]. Moreover, these families typically enjoy a higher socioeconomic status, mitigating some of the pressures faced by single-parent adolescents [32].

Notably, gender and age were also predictors of depression risk, consistent with existing research. Female adolescents and older adolescents were more susceptible to depressive symptoms [33]. Adolescent girls are more inclined to internalize emotions, and older adolescents experience greater psychological stress, both of which exacerbate depression symptoms [34, 35]. In single-parent families, the lack of stable support and additional family pressures may intensify these effects, increasing emotional distress among adolescents.

The nomogram model developed in this study holds significant clinical value. By integrating multiple factors, such as gender, family satisfaction, substance dependence, and sleep duration, the model effectively identifies high-risk single-parent adolescents, providing a basis for early screening and individualized interventions for mental health professionals. In practice, this model can be applied to mental health screenings in communities and schools to quickly identify high-risk individuals requiring additional support.

This study, while yielding meaningful results, has several limitations. First, the cross-sectional design limits causal inference, and longitudinal studies are needed to further validate the long-term effects of these risk factors on depression. Second, the reliance on self-reported data for both independent and dependent variables, including the CES-D for measuring depression, may introduce subjective bias and increase measurement error. Future studies should consider incorporating objective measurement tools to mitigate this bias. For instance, wearable devices could be used to track sleep duration and activity levels, smartphone data analytics could provide more precise usage patterns, and clinical assessments by mental health professionals could offer a more valid and reliable measure of depression.

Moreover, our study prioritized identifying predictors based on available data, limiting the exploration of alternative explanations. Key aspects such as the bidirectional nature of smartphone use and depression, unmeasured parenting influences, and pre-existing vulnerabilities within a diathesis-stress framework require further investigation.

Additionally, certain psychosocial factors, such as social support, coping mechanisms, and family history of depression, were not included in this study due to the limitations of the available questionnaire data. Future research should incorporate these variables to provide a more comprehensive understanding of depression risk among left-behind adolescents in single-parent families.Finally, the sample was mainly drawn from a specific region, and future studies should consider adolescents from different cultural backgrounds to enhance the model’s generalizability. Future research could explore additional potential factors influencing depression, such as social support and psychological resilience, to further refine risk prediction models.

## Conclusion

This study developed a nomogram-based model to assess depression risk among left-behind adolescents from single-parent families. By integrating demographic characteristics, family dynamics, health status, and lifestyle habits, the model effectively identifies high-risk individuals. Key risk factors included prolonged parental separation, substance dependence, inadequate sleep (both weekday and weekend), and chronic illness, while protective factors such as high family satisfaction and higher parental education were associated with a reduced risk of depression. The model demonstrated a high level of predictive accuracy, with strong discrimination between high and low-risk individuals, making it a practical tool for early screening and targeted interventions in clinical and community settings.

## Data Availability

The data that support the findings of this study are available at: 10.57760/sciencedb.12150.
